# Stakeholder Perspectives on Barriers and Facilitators for the Adoption of Virtual Clinical Trials: Qualitative Study

**DOI:** 10.2196/26813

**Published:** 2021-07-06

**Authors:** Romée Melanie Helena Coert, James Kenneth Timmis, André Boorsma, Wilrike J Pasman

**Affiliations:** 1 Department of Microbiology and Systems Biology Nederlandse Organisatie voor Toegepast Natuurwetenschappelijk Onderzoek Zeist Netherlands; 2 Faculty of Science Athena Institute Vrije Universiteit Amsterdam Amsterdam Netherlands

**Keywords:** virtual clinical trials, decentralized clinical trials, adoption, do-it-yourself, wearables, diffusion of innovation theory, clinical trials, digital health, virtual health

## Abstract

**Background:**

Conventional clinical trials are essential for generating high-quality evidence by measuring the efficacy of interventions in rigorously controlled clinical environments. However, their execution can be expensive and time-consuming. In addition, clinical trials face several logistical challenges regarding the identification, recruitment, and retention of participants; consistent data collection during trials; and adequate patient follow-up. This might lead to inefficient resource utilization. In order to partially address the current problems with conventional clinical trials, there exists the need for innovations. One such innovation is the virtual clinical trial (VCT). VCTs allow for the collection and integration of diverse data from multiple information sources, such as electronic health records, clinical and demographic data, patient-reported outcomes, anthropometric and activity measurements, and data collected by digital biomarkers or (small) samples that participants can collect themselves. Although VCTs have the potential to provide substantial value to clinical research and patients because they can lower clinical trial costs, increase the volume of data collected from patients’ daily environment, and reduce the burden of patient participation, so far VCT adoption is not commonplace.

**Objective:**

This paper aims to better understand the barriers and facilitators to VCT adoption by determining the factors that influence individuals’ considerations regarding VCTs from the perspective of various stakeholders.

**Methods:**

Based on online semistructured interviews, a qualitative study was conducted with pharmaceutical companies, food and health organizations, and an applied research organization in Europe. Data were thematically analyzed using Rogers’ diffusion of innovation theory.

**Results:**

A total of 16 individuals with interest and experience in VCTs were interviewed, including persons from pharmaceutical companies (n=6), food and health organizations (n=4), and a research organization (n=6). Key barriers included a potentially low degree of acceptance by regulatory authorities, technical issues (standardization, validation, and data storage), compliance and adherence, and lack of knowledge or comprehension regarding the opportunities VCTs have to offer. Involvement of regulators in development processes, stakeholder exposure to the results of pilot studies, and clear and simple instructions and assistance for patients were considered key facilitators.

**Conclusions:**

Collaboration among all stakeholders in VCT development is crucial to increase knowledge and awareness. Organizations should invest in accurate data collection technologies, and compliance of patients in VCTs needs to be ensured. Multicriteria decision analysis can help determine if a VCT is a preferred option by stakeholders. The findings of this study can be a good starting point to accelerate the development and widespread implementation of VCTs.

## Introduction

Developers of health interventions (eg, drugs, medical devices, diets, or procedures) have to demonstrate via clinical evidence that their technologies do no, or minimal, harm to patients and improve treatment outcomes [1,2]. Rigorous clinical research, and clinical trials specifically, are necessary to demonstrate sufficient efficacy and safety profiles in order for regulators to grant marketing authorizations and, in turn, for patients to benefit from the introduction of better treatments. However, the execution of a clinical trial is expensive and time-consuming. In addition, clinical trials face several logistical challenges that might lead to inefficient resource utilization [3,4]. These major challenges primarily pertain to the identification, recruitment, and retention of participants; attainment of informed consent; consistent data collection during trials; and adequate patient follow-up [4-6]. Moreover, several demographic groups such as ethnic minorities and elderly patients are underrepresented in clinical trials and therefore the generalizability of trial results might not be immediately clear [4,7,8]. In addition, real-life settings might sometimes be more suitable to comprehensively or appropriately assess the benefits (and risks) of health interventions. Consequently, the ecological validity—capturing data in a real-life setting that reflects the circumstances where the intervention will ultimately occur and across diverse populations—of conventional clinical trials might be limited [6,9]. In order to partially address the current problems with conventional clinical trials, there is a need for innovations in the clinical trial process.

A novel clinical trial concept is the digital clinical trial or virtual clinical trial (VCT), also known as a decentralized, do-it-yourself, remote, siteless, or innovative health trial [4,10,11]. VCTs can be defined as “trials executed through telemedicine, mobile/local healthcare providers and/or mobile technologies” [12,13]. VCTs allow for the collection and integration of diverse data from multiple information streams, such as electronic health records, clinical and demographic data, patient-reported outcomes, anthropometric and activity measurements, and data collected by digital biomarkers or (small) samples that participants can collect themselves (eg, blood droplet, saliva, or fecal sample sent for analysis to a lab) [4]. They could be used, for example, in dermatology research where skin diseases can be evaluated remotely [12]. In a study from Singer and colleagues [14], 69 participants could easily take photos of their skin after treatment and send them to a physician for evaluation. Furthermore, VCTs might also be useful in nutrition research where participants follow a diet and perform exercises or physical activity at home guided or monitored by health technologies such as mobile apps, wearables, online data collection, or web-based tests [15,16]. Such digital diagnostics and tools have to meet high validity and reliability quality standards that might be difficult to attain before they can be applied in these new types of trials [4]. In addition, data protection could be an issue since large amounts of sensitive health information might be transferred [12]. Nevertheless, the proper implementation of VCTs can reduce trial costs per participant by up to 50% (compared to conventional clinical trials) because many relevant tests can be performed and evaluated without the need for patients to visit specific sites [17-19]. This also means that less time from health care professionals is needed for data collection. Additionally, a substantially increased volume of data can be collected from patients’ daily environment and, thereby, potentially provide early estimation of intervention effectiveness [18,19]. Moreover, digital health technologies, such as wearables, can provide continuous monitoring of trial participants to rapidly identify adverse events [4]. Because, for example, recruitment and enrolment of patients, and (long-term) follow-up can be done remotely, the burden of patient participation is reduced, and participant diversity and retention can be significantly improved [10,20]. Importantly, VCTs can also deliver a more patient-centered approach and engage patients in lifestyle and clinical research, and thereby improve their overall health literacy [21]. Lastly, the recent COVID-19 pandemic further highlights the advantages of VCTs for various stakeholders: although clinical trial organizations had to put their traditional site trials on hold, virtual visits and data collection were still partially possible online [22,23]. As a consequence, Izmailova and colleagues [23] developed a decision tree for migration from clinic to remote activities.

Although VCTs have the potential to provide huge value to clinical research and, by extension, patients, VCT adoption is not commonplace as of yet [4]. This is surprising, as failing to explore all options of health intervention advancements runs the risk of missing opportunities to preserve and promote patients’ health and improve overall resource distribution in health care. Research into the perspectives of stakeholders such as research organizations, pharmaceutical organizations, and food and health organizations is needed. Previous research from the Clinical Trials Transformation Initiative (CTTI) was focused on the perspectives of sponsors to identify legal and regulatory challenges of VCTs, but research organizations and food and health organizations were not included [13]. Therefore, the aim of this study was to better understand barriers and facilitators to the adoption of VCTs by determining the factors that influence individuals’ considerations regarding VCTs from the viewpoints of pharmaceutical organizations, food companies, and a research organization, by taking a research organization’s perspective. The research organization Nederlandse Organisatie voor Toegepast Natuurwetenschappelijk Onderzoek (TNO; Netherlands Organisation for Applied Scientific Research) is an independent, government-funded applied research organization that aims to nurture innovation by closing the gap between research and industry. TNO connects companies and knowledge in order to create innovations that sustainably strengthen the competitiveness of the high tech sector and, in turn, improve the well-being of society [24]. This study was a first pilot investigation to examine the readiness of companies and organizations to adopt VCTs.

## Methods

### Study Overview

We conducted a qualitative study on the perspectives of stakeholders about the adoption of VCTs. The Consolidated Criteria for Reporting Qualitative Research (COREQ) checklist for reporting qualitative research was followed (Multimedia Appendix 1) [25]. Approval for the qualitative study was granted by the Internal Review Board of TNO in April 2020 (reference number: 2020-034).

### Theoretical Considerations

To base our investigation on a solid theoretical framework, we utilized Rogers’ diffusion of innovation theory. Rogers’ framework aims to explain how and why a new innovation or technology is, or is not, adopted, where adoption can be defined as “the full use of an innovation [when the innovation] is the best course of action available” [26]. A VCT can be considered a process innovation, which is defined as the implementation of a new or improved delivery method, including substantial changes in techniques, equipment, and/or software [27]. Zhang and colleagues [28] reported their investigation of the factors leading to a successful eHealth process innovation. They used Rogers’ theory to analyze patient acceptance and identify reasons for the utilization of eHealth innovations [28]. Several other recent studies have also used Rogers’ framework to better understand health technology adoption [28,29]. Therefore, Rogers’ theory was considered to be an appropriate framework for this study. Multimedia Appendix 2 [30-33] provides more details about Rogers’ theory and concepts, including the five stages of the innovation-decision process.

### Data Sources and Sampling Strategy

A purposive sampling strategy was used to select invitees [25]. Three stakeholder groups were approached via email: invitees were recruited from TNO (within which people working in the health division were approached), pharmaceutical organizations, and food and health organizations. Within TNO, the Microbiology and Systems Biology (MSB) department supports the research and development activities of companies in the agriculture and food, health, personal care, chemistry, biotechnology, and pharmaceutical sectors. The department collaborates with pharmaceutical, food, and health organizations as well as other companies to set up clinical trials [24]. Within the pharmaceutical industry, a distinction between sponsors and contract research organizations (CROs) can be made. Food and health companies can be defined as organizations that aim to improve health by healthy foods. These organizations study nutrition by conducting nutrition trials.

Twenty potential participants received an interview invitation. Due to the COVID-19 pandemic, all interviews were held by telephone or Skype. Invitees were enrolled in the study if they met the following inclusion criteria: speak Dutch or English fluently; work for one of the three stakeholder groups (research organization, pharmaceutical industry, or a food company) in a key position of clinical trial execution; have experience and/or interest in using VCTs; and are willing and able to participate and sign an online informed consent form.

### Data Collection and Instrument

The semistructured interviews were carried out by author RMHC. The interview guide consisted of themes and questions based on the five innovation stages as suggested by Rogers, namely: knowledge, persuasion, decision, implementation, and confirmation (Multimedia Appendix 3). The interview guide was discussed with and approved by all authors. Furthermore, the interview was piloted with 2 independent researchers at TNO [25]. Once the invitees had given consent, the interviews were conducted and audio recorded. Field notes were made during and directly after the interviews.

### Data Analysis

The audio recordings were transcribed immediately after the interview, new concepts were identified, and the degree of data saturation was measured [34-37]. In addition, to confirm the accuracy of content and key messages, participants were asked to review a brief summary of the interview and a couple of quotes, and comment if necessary [35,37]. The recordings were deleted directly after transcription, and the transcripts were stored as Microsoft Word files (Microsoft Corp) on a secured laptop per proper data and privacy protection measures. The data were analyzed by using the thematic analysis method suggested by Clarke and Braun [38], which consists of the following 6 steps: familiarization with the data, initial code generation, theme identification, theme review, theme definition and naming, and, finally, report production [38]. The data obtained from the interviews were coded by RMHC and merged into a code book. A 5% check (1 interview transcript and coding) was done by another researcher to ensure that coding was performed consistently. The data from the interviews were descriptively analyzed with Microsoft Excel 2016 (Microsoft Corp).

## Results

### Participant Characteristics

From the 20 potential participants who were approached, 4 individuals were excluded from this study; 2 did not respond to the invitation, and 2 were not experienced enough in VCTs and thus did not met the inclusion criteria. In total, 16 individuals were interviewed between April and June 2020. Six employees from a research organization, 6 pharmaceutical employees, and 4 individuals from food companies were enrolled in the study. Among the pharmaceutical participants, one was an employee of a CRO. This person was grouped into the pharmaceutical cohort since the results of the interviews between the CRO and pharmaceutical companies were much aligned. All interviewees from the research organization lived in The Netherlands, while 2 pharmaceutical interviewees and 2 interviewees from food companies were based in Ireland, Denmark, France, and Germany (Table 1). In Table 2, the type of organizations where interviewees were employed is shown.

**Table 1:** Participant characteristics (N=16).

Characteristic			Participants, n (%)
**Organization**			
	**Research organization**		6 (37.5)
		Junior	0 (0)
		Senior^a^	6 (37.5)
	**Pharmaceutical companies**		6 (37.5)
		Junior	2 (12.5)
		Senior	4 (25.0)
	**Food companies**		4 (25.0)
		Junior	2 (12.5)
		Senior	2 (12.5)
**Country**			
	The Netherlands		12 (75.0)
	Other parts of Europe (Ireland, Denmark, France, and Germany)		4 (25.0)
**Gender**			
	Male		13 (81.25)
	Female		3 (18.75)

^a^A senior has more than 5 years of experience in his or her job.

**Table 2:** Type of organizations where interviewees were employed (N=11).

Organization type	Organizations included, n (%)
Research organization	1 (9.1)
Pharmaceutical companies	6 (54.5)
Food companies	4 (36.4)

Of the 6 individuals affiliated with the research organization, 5 were business developers. Business developers were included since they are the initial point of contact with business partners and discuss different options for conducting studies. They have ideas about how VCTs could be interesting for their clients and whether the research organization should be involved in VCTs. The remaining individual was a senior scientist conducting clinical research in collaboration with pharmaceutical companies and universities.

The 6 participants from the pharmaceutical companies had various professional backgrounds. Two were country study managers leading a team that conducts clinical trials. Moreover, they are responsible for the quality of the research. Two participants worked within an innovation department. One was the medical lead of innovation, and the other worked as the head of clinical technology and innovation. The medical lead of innovation was responsible for several (clinical trial) innovation projects within the company. The head of clinical technology and innovation was closely involved with conducting VCTs. Another interviewee was a clinical scientist, who was involved in conducting clinical trials. The last participant was a medical advisor and data scientist, and was responsible for coordinating clinical research and analyzing clinical trial data.

**Figure 1:**
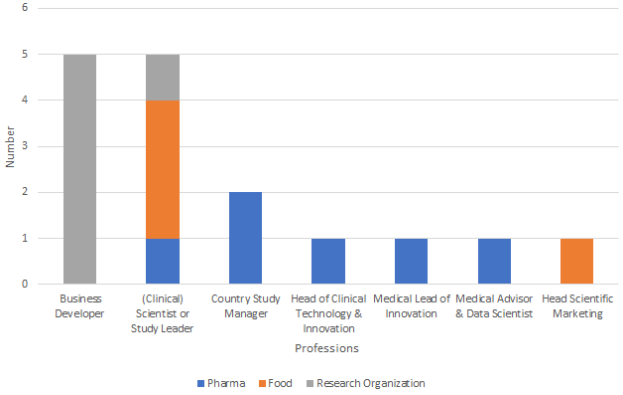
Overview of participants’ professions.

Three of the 4 interviewees from the food companies were (clinical) scientists and/or study leaders. They were responsible for conducting or leading clinical trials. The remaining participant was head of scientific marketing within a food company, who conceptualized, planned, and implemented clinical research. Figure 1 displays the professional backgrounds of the participants.

A total of 12 interviews were conducted by telephone and 4 by Skype (with video). Interviews lasted approximately 45 minutes. After the 15th interview, data saturation was reached. Data collection was continued for one more interview to confirm that no new concepts were mentioned.

### Overview of Findings

Table 3 illustrates current barriers and facilitators, according to stakeholder perspectives.

**Table 3:** Barriers and facilitators for the adoption of virtual clinical trials. Counts indicate how many interviewees agreed on certain barriers and facilitators.

Barriers and facilitators		Research organization (n=6), n (%)	Pharmaceutical companies (n=6), n (%)	Food companies (n=4), n (%)	Total across all organization types, n (%)
**Barriers**					
	Acceptance by regulatory authorities	3 (18.75)	3 (18.75)	2 (12.5)	8 (50.0)
	Technical issues (standardization, validation, and data storage)^a^	4 (25.0)	4 (25.0)	1 (6.25)	9 (56.3)
	Compliance and adherence^a^	5 (31.25)	6 (37.5)	3 (18.75)	14 (87.5)
	Lack of knowledge or understanding	2 (12.5)	1 (6.25)	3 (18.75)	6 (37.5)
**Facilitators**					
	Involving regulators in the development process	3 (18.75)	3 (18.75)	1 (6.25)	7 (43.8)
	Exposure to the results of pilot studies^a^	5 (31.25)	2 (12.5)	3 (18.75)	10 (62.5)
	Clear instructions and assistance for patients^a^	4 (25.0)	6 (37.5)	3 (18.75)	13 (81.3)

^a^Barriers and facilitators that many stakeholders agreed on (ie, more than half [>8] of the interviewees mentioned it).

### Barriers to VCT Adoption

#### Acceptance by Regulatory Authorities

The concern of many participants was that regulatory authorities such as the Food and Drug Administration (FDA) and European Medicines Agency are not ready to accept VCTs because the procedures to evaluate VCTs are currently not standardized or well established. One person from a pharmaceutical company mentioned that, although some guidelines were available, they were vague and abstract, and this made it highly challenging to estimate if a VCT would be approved. For the pharmaceutical industry, this was important as evidence would have to be obtained by approved methods; otherwise pharmaceutical products cannot receive approval.

#### Technical Issues: Standardization, Validation, and Data Storage

Participants mentioned several technical issues regarding the execution of VCTs. One of them was the lack of standardization. Participants expressed that the development of smart devices is fast-paced and, in addition, the algorithms of these devices are not always known, which could lead to these devices being considered a “black box.” Therefore, respondents explained that the data collected today would become outdated in a year’s time as new algorithms and smart devices become available. In addition, investigators are mandated to store study data for 15 years, but if the technology becomes outdated and cannot be updated, data storage (and access) can be problematic. Another issue mentioned was accessibility of data through an app or wearable developer, whereby the researcher has no access to the propriety data. Respondents were also concerned about the accuracy of wearables; although many wearables are currently available, most have not been validated. If a wearable did not have formal validation, the data it generated could not be used as clinical evidence.

#### Compliance or Adherence

Respondents from all organization types mentioned that even in normal clinical trials, compliance (the degree to which a patient correctly follows an intervention) was an issue. In a VCT, they believed that this could become an even bigger issue, as in-person contact with participants is limited. For example, the drug would have to be taken, or the wearable used, by the trial participant only, which is challenging to monitor in a decentralized setting:

If you conduct it [a trial] in a clinical setting, there is someone [a study nurse/researcher] standing next to the participant, so he or she checks if everything is done correctly. But if you do it [ie, a trial] at home ... you [a researcher] don't see it. Or, for example, you [a researcher] track a mobile phone, it can also remain on the table, in another room.

P3

#### Lack of Knowledge or Understanding

The interviews revealed that some organizations, mainly food companies and the research organization, had a lack of knowledge regarding the opportunities offered by VCTs. Parts of clinical trials could be done remotely, but food companies and some interviewees from the research organization were not familiar with conducting clinical trials remotely and/or the development of remote technologies. For example, the concrete utility of, and how to develop, VCTs remained unclear for food companies, as they were not aware of other organizations that utilize VCTs and did not know how to obtain relevant information. Therefore, they were more reluctant to explore VCTs. This indicates that collaboration between stakeholders, especially between food companies and the pharmaceutical industry, were not effective. Pharmaceutical companies, on the other hand, were aware of the opportunities offered by VCTs and knew that others were exploring this field and developing new platforms. Participants from all stakeholder groups mentioned that some patient groups might face various issues related to VCTs. In particular, elderly patients may not have the experience that is required, such as knowing how to use a smartwatch or the internet, for this study type, even though it would be highly preferable to include this patient group in trials. Consequently, a relevant part of the population might not be included in VCTs, which could lead to biased data and thus imprecise estimates of intervention effectiveness.

### Facilitators of VCT Adoption

#### Involving Regulators and Other Stakeholders in Development Processes

According to participants, the acceptance of VCTs could be facilitated by involving regulators early in the development phase of a VCT. Furthermore, it was also important to collaborate with other stakeholders during this phase such as other pharmaceutical companies, research organizations, and food companies, in addition to also incorporating the clinic (doctors and patients) to investigate their needs:

About 15 years ago, pharmaceutical companies were loose [isolated] strongholds. But now you see more and more that the companies are working together [open innovation]. And I think collaboration is the future. It cannot be otherwise because the development of new innovative medicines is very expensive. You just have to do it together.

P1

#### Exposure to the Results of Pilot Studies

According to the pharmaceutical participants, many of them were currently doing validation or pilot studies with wearables, online platforms (eg, for trial recruitment), or mobile apps. Conducting such pilot studies and publishing the results would support other organizations in understanding VCTs and how to use them. It might also convince regulators that the collected data were reliable and added value to new health interventions.

#### Clear Instructions and Assistance for Patients

All three stakeholder groups indicated that patients in a VCT needed additional assistance from the researcher or study nurse with, for instance, the proper use of a wearable. One participant (pharmaceutical company) indicated that guidance was needed because the tool (eg, wearable) itself could be useless without user input. Specific groups such as elderly trial participants need proper instructions and guidance (eg, via a helpdesk).

### The Decision to Adopt VCTs

A point mentioned by participants across all organizations was that adoption depended on the type of study to be performed. In principle, if a research question was suitable, for instance, if a researcher was interested in studying lifestyle changes measured through a wearable or collected via an online questionnaire at home, a VCT could provide a significantly richer data set (including real-life data) when compared to a conventional clinical trial. On a more practical note, for many respondents, the decision to adopt VCTs also depended on the costs of the trials. If VCTs could in effect reduce costs, some respondents would be inclined to conduct a VCT rather than a conventional clinical trial.

### Options to Apply VCTs

The three stakeholder groups made several suggestions on what types of tools could be used and how VCTs could be implemented. Contemporary smart devices or wearables could be used to measure health parameters (eg, heart rate, temperature, oxygen saturation, respiration rate, blood pressure, blood glucose level) and monitor patients while the investigational intervention or drug is studied. Next to noninvasive measurements, the collection of small samples, such as drawing blood by a finger prick, is also possible. By extension, multiple blood droplet analysis devices exist that enable consumers to collect data (eg, blood cholesterol, glucose, or ferritin) themselves and at home. Furthermore, participants mentioned the possibility of conducting online tests consisting of questionnaires or cognition assessments that could be used in, for example, nutrition studies, where participants consume a daily dietary supplement. The effect of the supplement on satiety or gastrointestinal function could be monitored easily online and does not require clinical assistance.

**Figure 2:**
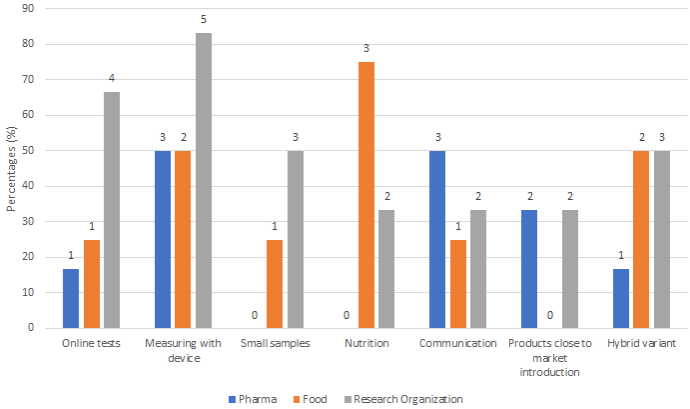
Options related to virtual clinical trials (VCTs) that interviewees agreed on. The counts and percentages provide an indication of how many participants suggested VCTs for a specific study type.

Communication between patients and researchers/doctors could be executed virtually (ie, online). Visits to the clinic, which usually would be planned in conventional clinical trials, could be done remotely, especially for follow-up visits once the patient is considered disease-free. Respondents from the pharmaceutical companies and the research organization explained that a VCT could be implemented when a product or service was close to market introduction. Such trials might investigate how the product or service works in the real-life environment of patients. The hybrid variant was mentioned as the most promising form of a VCT. This means that such trials would have virtual and conventional clinical trial components. For instance, conventional clinical trials could have a hybrid locality approach by collecting some data outside of the central research facilities (eg, plasma and tissue samples are collected by affiliated labs and sent to central locations for testing). Figure 2 shows agreement among the interviewees about potential VCT options.

## Discussion

### Principal Results

In this study, participants acknowledged the innovative value of VCTs and agreed that the development of VCTs should be further explored and promoted. However, various aspects have to be improved before VCTs can be widely adopted, such as improving effective collaboration between all stakeholders, clinically validating smart devices and wearables for data collection, and ensuring patient compliance. The data obtained from our interviewees demonstrate that pharmaceutical companies and the research organization in particular are aware of the benefits and disadvantages of VCTs. However, the applicability of VCTs in research is not yet well known. Therefore, informing stakeholders of the advantages of VCTs is an important factor to speed up their consideration and adoption. This is relevant especially now during the COVID-19 pandemic, due to which many conventional randomized controlled trials have been halted; here, VCTs can make a substantial contribution to the continuation of certain types of crucial clinical research [22].

### Comparison With Prior Work

#### Collaboration

All participants agreed on the importance of enhancing collaboration among stakeholders and, in particular, with regulatory authorities. The acceptance of VCTs by regulators was perceived as a barrier, which is in line with previous research. Polhemus et al [39] studied the adoption of patient-facing technologies in clinical trials from an industry perspective, and one of the main barriers they identified were regulatory challenges. This was due to a lack of specific guidance (no or few regulations), geographic variability of guidance, and internal perceptions and misperceptions [39]. In addition, the CTTI stated that telemedicine was not widely used in the design and conduct of clinical trials because of legal and regulatory considerations [40]. Due to the COVID-19 pandemic, medical centers and CROs are required to set up online communication and information repositories, resulting in the release of new VCT guidelines by the FDA [22,41]. When designing VCTs, it is important to take these guidelines into consideration. The data infrastructure, processing, analysis, and interpretation of a VCT are different compared to a conventional clinical trial. As also proposed by the CTTI, there is a need for collaboration across various experts such as CROs, pharmaceutical organizations, research organizations, and food and health organizations on one hand, and the companies developing new technologies (eg, wearables) on the other [13,42,43].

#### Validation of Data Collection Tools

All participants emphasized that the accuracy of the data collected from, for example, wearables; do-it-yourself blood analysis of glucose, cholesterol, hemoglobin, etc; or blood pressure devices in VCTs, is crucial. Currently, there exist many consumer-grade devices that promise to improve health and wellness without scientific evidence substantiating such claims; hence, there is an urgent need for clinically validated devices and wearables [9,43]. The quality assurance (and measurement accuracy) of technologies has been questioned before and several challenges were identified by previous studies [39,44]. The findings of Abdolkhani et al [44] indicated that technical and policy standards need to be developed to guarantee the quality of data generated from wearables. Besides the need for such standards, it is important to validate the wearables by “fit-for-purpose validation” [45]. In such trials, it should become clear, within a very short time frame, if the technology is suitable to accurately measure clinically meaningful endpoints. According to Goldsack et al [45], the evaluation of a wearable device, or a biometric monitoring device, should consist of a three-component framework, consisting of verification, analytical validation, and clinical validation [45]. This has to be completed before technologies can be utilized in VCTs.

#### Compliance

As described by our interviewees, investigators must carefully consider how to ensure a high degree of compliance in VCTs. Moreover, they explained that proper instructions are crucial as patients have to fully understand what is expected of them as active partners in a trial and need to know how to obtain information. A clear and simple protocol is therefore required [46]. According to previous research, participants who understand the expectations of a clinical trial are more willing and able to comply [47]. Furthermore, some participant groups, such as the elderly can be less literate regarding electronic and smart devices [48,49]. In order to overcome this challenge, interviewees suggested that these participant groups receive more assistance during VCTs. The technology used in a VCT needs to be user-friendly, and the trial itself needs to be as simple as possible [48]. Therefore, it has been recommended by the CTTI that participants be engaged in technology selection. Only technologies that are easy to learn, simple, convenient to use, and physically comfortable should be included in a VCT [46,50].

### Implications for Practice and Policy

In order to improve the adoption of VCTs, it is important that all stakeholders collaborate with each other [51]. One option for enhancing collaboration among all stakeholders and exploring if a VCT is a preferred option is an multicriteria decision analysis (MCDA) [52]. Although we have not yet implemented this method, an MCDA might be a good option for researchers who are considering VCTs as a data collection tool. An MCDA is a set of techniques that can help decision-makers take into account and integrate multidimensional data (eg, attributes of benefit) and rank different decision alternatives [52]. For example, the preferences of different criteria or parameters relevant to clinical trials could be elicited a priori and subsequently “preloaded” as preference profiles into dedicated MCDA software. This would potentially result in a more transparent and rapid decision process, and more effective support for decision-makers in determining if a VCT, or some of its components, would be beneficial for their trial. Although an MCDA gives a transparent methodology to compare different decision alternatives, it is an extensive technique that might not be applicable to every context; one setting might be more appropriate for an MCDA than another. For instance, it could be more easily implemented in larger organizations with, in relative terms, larger budgets than in smaller organizations. Next to the MCDA, stakeholders should invest in the use of validated diagnostics to obtain the most reliable results in VCTs [45]. The adherence of patients can be improved by properly devising tools that can motivate them (eg, in a playful way, that is, gamification) or via online monitoring. Because a VCT is a new form of clinical trial with a digital approach, additional assistance might be necessary for some participants. For this reason, we suggest the installation of helpdesks to support trial participants by answering their questions and providing them with support 24/7.

### Strengths, Limitations, and Future Research

Underpinned by a well-known theoretical framework of innovation, our qualitative research approach allowed us to elicit and characterize the broad experiences of interviewees with the VCT adoption process [35]. Furthermore, we made use of the COREQ checklist and an interview guide, which improved the validity and standardization of the interviews, while allowing improvised follow-up questions based on our interviewees’ responses [25,35]. Whereas most studies concerning VCTs are conducted in the United States, our research focused on Europe, which is unique.

The conduct of this research in the European Union could be framed as a strength but also as a limitation. Data sampling was restricted to Europe, mostly to The Netherlands. Since the United States is more advanced in conducting VCTs, follow-up studies should also include this country in their data sample. Furthermore, this pilot study only included pharmaceutical companies (including a CRO), food companies, and a single research organization. Health care professionals, patients or participants, regulatory authorities, CROs, and payers are also important stakeholders in VCTs. According to previous studies, these stakeholders should also be included in the process of clinical trial development [42,53]. Therefore, future research should focus on these stakeholders, and their preferences, value points, and perspectives on VCTs. Lastly, inclusion of only one research organization introduced selection bias, leading to a less representative study sample.

### Conclusions

This study used a qualitative research approach to identify the barriers and facilitators behind the adoption of VCTs and explored how this process can be improved. Collaboration among all stakeholders in VCT development is essential to increase knowledge and awareness. Organizations should invest in accurate data collection technologies, and compliance of patients in VCTs needs to be ensured. Furthermore, we suggest conducting an MCDA to explore whether a VCT is a preferred option by stakeholders; this can considerably enhance the decision-making process. The findings of this study can be a good vantage point to accelerate the development and widespread implementation of VCTs.

## Appendix

Multimedia Appendix 1 COREQ checklist.

Multimedia Appendix 2 Rogers’ diffusion of innovation theory.

Multimedia Appendix 3 Interview guide.

## Abbreviations

COREQ: Consolidated Criteria for Reporting Qualitative Research
CRO: clinical research organization
CTTI: Clinical Trials Transformation Initiative
FDA: Food and Drug Administration
MCDA: multicriteria decision analysis
TNO: Nederlandse Organisatie voor Toegepast Natuurwetenschappelijk Onderzoek
VCT: virtual clinical trial
