# Supplementary figures and images for: Stakeholder Perspectives on Barriers and Facilitators for the Adoption of Virtual Clinical Trials: Qualitative Study
Source: J Med Internet Res. 2021 Jul 6;23(7):e26813. doi: 10.2196/26813 (PMC8294122; doi:10.2196/26813)

## Multimedia Appendix 1: COREQ checklist


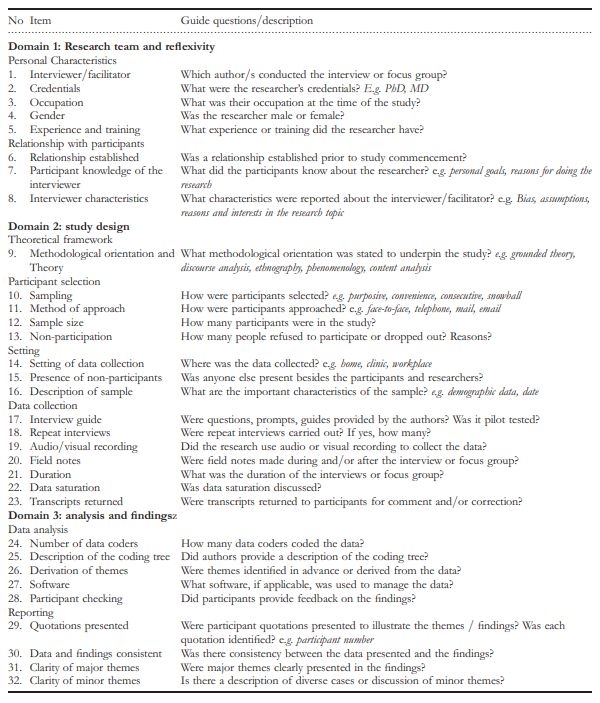

Supplement: Multimedia Appendix 1 [file jmir_v23i7e26813_app1.docx]
