# Supplementary material for: Stakeholder Perspectives on Barriers and Facilitators for the Adoption of Virtual Clinical Trials: Qualitative Study
Source: J Med Internet Res. 2021 Jul 6;23(7):e26813. doi: 10.2196/26813 (PMC8294122; doi:10.2196/26813)
Supplement: Multimedia Appendix 2 [file jmir_v23i7e26813_app2.docx]

## Multimedia Appendix 2 Rogers’ Diffusion of innovation theory

*‘Rogers’ Diffusion of innovation theory’* was used as a theoretical framework [50]. Here, further information about the theory and its’ applicability is provided.

Within Rogers’ innovation theory, there are four main elements that lead to the diffusion of an innovation: innovation, communication channels, time and the social system. An innovation can be described as an idea, practice or process that is perceived as novel by a person or other adoption entity. Communication channels are needed to share information between adopters and time dimension should be considered. Because the diffusion of an innovation occurs in a social system, it could be influenced by the social system and might not be compatible with the current discourse within certain professions, fields and decision making bodies [26].

The innovation-decision process can be divided into five stages, as shown in Figure 3. In this process, an individual is motivated to seek information and process the information to decrease the uncertainty about the advantages and disadvantages of an innovation [26].


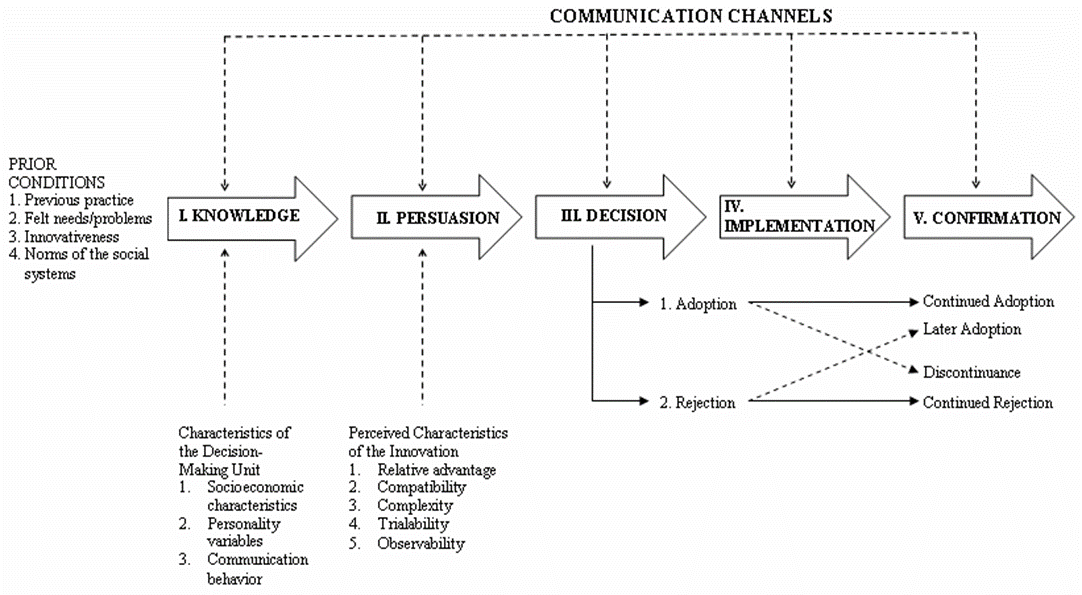


**Figure 3.** The five stages of the innovation-decision process [26].

The first stage is *knowledge*. In this stage, individuals or other units of adoption receive information about the innovation, namely: what the innovation is and how it works. This results in three types of knowledge: awareness-knowledge (existence of the innovation), how-to-knowledge (how to use the innovation), and principles-knowledge (why an innovation works) [26].

The second stage is *persuasion*, which occurs when an individual has a negative or positive attitude towards the innovation. Here, the perceived characteristics (adoption factors) of the innovation are relevant [26]. Adoption means “the full use of an innovation is the best course of action available” and rejection means “not to adopt an innovation” [26,51]. In order to explain the adoption of an innovation, five characteristics that effect adoption are proposed: (1) *relative advantage*, (2) *compatibility*, (3) *complexity*, (4) *trialability* and (5) *observability* [52,53]*.* Below they will be explained:

1. *Relative advantage* is defined as “the degree to which an innovation is perceived as being better than the idea it supersedes” [50]. Rogers suggests that when innovations have a clear advantage over the previous innovation or idea, it will be more easily adopted and implemented [26]. ]
2. Compatibility can be defined as “the degree to which an innovation is perceived as consistent with the existing values, past experiences, and needs of potential adopters” [26]. Therefore, the innovation should be meaningful to the potential adopter. If the compatibility of the innovation is high, the likelihood of adoption, in turn, is higher [52].
3. *Complexity* is defined as “the degree to which an innovation is perceived as relatively difficult to understand and use” [50]. In contrast to the other adoption factors, complexity is negatively associated with the adoption rate. Consequently, complexity is an obstacle in the adoption of an innovation. Moreover, Rogers suggested that new innovations can be categorized on a complexity-simplicity scale. Sometimes, the content of the innovation may not be understood by potential adopters. When users perceive innovations as easy to use, the innovations will be more easily adopted [26].
4. *Trialability*, is defined as “the degree to which an innovation may be experimented with on a limited basis” [50]. The adoption of new innovations costs time, energy and other recourses. Therefore, innovations that can be tried before are more easily adopted [26].
5. And lastly, *observability* is defined as “the degree to which the results of an innovation are visible to others” [50]. In the process of adopting an innovation or technology, peer observation or role modelling is an essential motivational factor [26]. If there are observable positive results from the innovation, then the innovation tends to be adopted more easily [52].

The next stage is *decision*, in which the person chooses to adopt or reject the innovation. Adoption can be divided into a continued adoption or discontinuance decision. The latter means that the innovation is rejected after adopting it in an earlier stage. Furthermore, rejection is separated into continued rejection and later adoption. When an innovation is rejected at first, it can be adopted in a later stage [26].

In the *Implementation* stage, the innovation is put into practice. However, there could still be uncertainty about the outcomes of the innovations. Therefore, the implementer might need assistance from others to reduce the uncertainty about the outcomes [26].

The last stage is *Confirmation*. The innovation-decision has already been made, but in this stage, the person searches for support for his or her decision and this means that the attitude of the person is important. Based on the support for adoption and the attitude of the person, he or she chooses for discontinuance or later adoption [26].
