# Supplementary material for: Stakeholder Perspectives on Barriers and Facilitators for the Adoption of Virtual Clinical Trials: Qualitative Study
Source: J Med Internet Res. 2021 Jul 6;23(7):e26813. doi: 10.2196/26813 (PMC8294122; doi:10.2196/26813)
Supplement: Multimedia Appendix 3 [file jmir_v23i7e26813_app3.docx]

## Multimedia Appendix 3: Interview Guide

**Introduction**

**General: Background information**

- Could you briefly introduce yourself (name & current position)?
- Why are you interested in VCTs?

**Theme: Compatibility**

- What are your experiences with VCTs within your company?
- What factors do you think are important when carrying out VCTs?
- How could the virtual aspects of VCT improve the research within your work area?

**Theme: Relative advantage**

- What do you think are the advantages (positive outcomes) that could be accrued by introducing VCTs?
- What do you think are the disadvantages (unwanted events) of VCTs?
- Which types of interventions would be suited for VCTs?

**Theme: Complexity**

- Do you perceive VCTs methods as easy to understand and use?
- Which barriers do you see for applying VCTs on a broader scale?

**Theme: Observability**

- Do you know other companies in the food & health or pharma that use VCTs?
- What is remarkable about them?

**Theme: Implementation**

- Do you think researchers need additional assistance when VCTs are being implemented?
- How do you know that the quality of the measurements within a VCT is sufficient?

**Theme: Decision**

- On what basis would you decide to continue to use (adopt) VCT’s?
- On what basis would you decide to not to use (reject) VCT’s?
- Which parts of a clinical study can or should be carried out online?

**Closing**
